# CHINESE OLDER ADULTS’ PREFERENCES FOR INVOLVEMENT IN END-OF-LIFE CARE DECISIONS: CAUSES AND OUTCOMES

**DOI:** 10.1093/geroni/igae098.1748

**Published:** 2024-12-31

**Authors:** Yifan Lou, Jinyu Liu, Deborah Carr, Yaolin Pei

**Affiliations:** Virginia Commonwealth University, New Haven, Virginia, United States; Baylor University, Waco, Texas, United States; Boston University, Boston, Massachusetts, United States; University of Texas at Austin, Texas, United States

## Abstract

Understanding the end-of-life decision making styles of Chinese older adults is an important goal, especially against a backdrop of global population aging. Guided by the Shared Decision-making framework, we use data from 571 adults ages 50+ in Shanghai, China to develop statistically and conceptually distinct profiles of shared, delegated, and autonomous end-of-life decision making; and identify the psychosocial and health correlates of these profiles, and the impact of profile type on advance care planning. We use latent class analysis (LCA) to develop decision-making profiles capturing preferences for involvement in information exchange, deliberation, and decisional control in end-of-life decision-making. Four profiles emerged. Delegated decision makers (16%) almost all prefer to have family or doctors make their treatment and care placement plans. Lead decision makers (49%) want to take the lead in information exchange and final decisions, although they value collaborative discussions. Acquiescent decision makers (15%) prefer to follow their family’s wishes, if discrepant preferences exist. Autonomous decision makers (19%) prefer not to engage others in any process. Older age is associated with delegated decision making, whereas higher income and education are associated with autonomous decision-making. Acquiescent decision makers are less likely to have thought about or discussed their end-of-life wishes, despite their preferences for sharing decisions. Our results reveal great diversity in older adults’ approaches to autonomous versus shared end-of-life decision making, and thus health care providers must devise tailored approaches for working with older patients and their families.

